# *catena*-Poly[[diaqua­(1,2,3-benzothia­diazole-7-carboxyl­ato-κ*O*)copper(II)]-μ-1,2,3-benzothia­diazole-7-carboxyl­ato-κ^2^ *N* ^2^:*O*]

**DOI:** 10.1107/S1600536809032036

**Published:** 2009-09-09

**Authors:** Guo-Ying Zhang, Xin Zhang, Hai-Zhen Xu

**Affiliations:** a College of Chemistry and Life Science, Tianjin Normal University, Tianjin 300074, People’s Republic of China

## Abstract

In the polymeric title complex, [Cu(C_7_H_3_N_2_O_2_S)_2_(H_2_O)_2_]_*n*_, the Cu^II^ centre is surrounded by three 1,2,3-benzothia­diazole-7-carboxyl­ate and two water mol­ecules. A 1,2,3-benzothia­diazole-7-carboxyl­ate ligand bridges two Cu^II^ centres, with a Cu⋯Cu distance of 9.006 (2) Å. The four O atoms in the equatorial planes around each Cu^II^ centre form a distorted square-planar arrangement, while the distorted square-pyramidal coordination is completed by the symmetry-related N atoms of the bridging 1,2,3-benzothia­diazole-7-carboxyl­ate ligands. In the crystal structure, inter­molecular O—H⋯O and O—H⋯N hydrogen bonds link the mol­ecules into a three-dimensional supra­molecular network.

## Related literature

For general background, see: Addison *et al.* (1984 ▶); Hou *et al.* (2004 ▶); Lan *et al.* (2009 ▶); Wang *et al.* (2008 ▶). For related structures, see: Batzel & Boese (1981 ▶); Fan *et al.* (2005 ▶); Lukashuk *et al.* (2007 ▶); Qin *et al.* (2009 ▶); Richardson & Steel (2002 ▶); Walter & Beat (1997 ▶). 
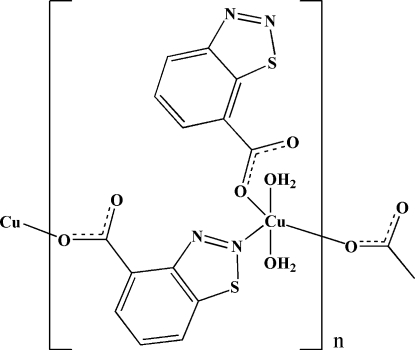


## Experimental

### 

#### Crystal data

[Cu(C_7_H_3_N_2_O_2_S)_2_(H_2_O)_2_]
*M* *_r_* = 457.95
Triclinic, 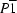

*a* = 9.0061 (18) Å
*b* = 9.4989 (19) Å
*c* = 11.274 (2) Å
α = 86.62 (3)°
β = 70.00 (3)°
γ = 76.69 (3)°
*V* = 881.8 (4) Å^3^
*Z* = 2
Mo *K*α radiation
μ = 1.52 mm^−1^
*T* = 294 K
0.28 × 0.26 × 0.24 mm

#### Data collection

Rigaku R-AXIS RAPID-S diffractometer
Absorption correction: multi-scan (*SADABS*; Bruker, 1998 ▶) *T*_min_ = 0.676, *T*_max_ = 0.712
7632 measured reflections
3089 independent reflections
2767 reflections with *I* > 2σ(*I*)
*R*_int_ = 0.027

#### Refinement

*R*[*F* ^2^ > 2σ(*F* ^2^)] = 0.036
*wR*(*F* ^2^) = 0.083
*S* = 1.08
3089 reflections
244 parameters
H-atom parameters constrained
Δρ_max_ = 0.72 e Å^−3^
Δρ_min_ = −0.70 e Å^−3^

### 

Data collection: *CrystalClear* (Rigaku/MSC, 2005 ▶); cell refinement: *CrystalClear*; data reduction: *CrystalClear*; program(s) used to solve structure: *SHELXS97* (Sheldrick, 2008 ▶); program(s) used to refine structure: *SHELXL97* (Sheldrick, 2008 ▶); molecular graphics: *SHELXTL* (Sheldrick, 2008 ▶); software used to prepare material for publication: *SHELXTL*.

## Supplementary Material

Crystal structure: contains datablocks I, global. DOI: 10.1107/S1600536809032036/hk2737sup1.cif

Structure factors: contains datablocks I. DOI: 10.1107/S1600536809032036/hk2737Isup2.hkl

Additional supplementary materials:  crystallographic information; 3D view; checkCIF report

## Figures and Tables

**Table 1:** Selected geometric parameters (Å, °)

Cu1—O4^i^	1.9405 (19)
Cu1—O1	1.945 (2)
Cu1—O1*W*	1.991 (2)
Cu1—O2*W*	1.980 (2)
Cu1—N4	2.311 (2)

Symmetry code: (i) 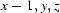.

**Table 2:** Hydrogen-bond geometry (Å, °)

*D*—H⋯*A*	*D*—H	H⋯*A*	*D*⋯*A*	*D*—H⋯*A*
O1w—H1wA⋯N3^ii^	0.85	2.02	2.859 (4)	169
O1w—H1wB⋯N1^iii^	0.85	2.12	2.957 (4)	170
O2w—H2wA⋯O3^iv^	0.85	1.84	2.680 (3)	172
O2w—H2wB⋯O2^v^	0.85	1.84	2.684 (3)	172

Symmetry codes: (ii) 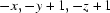; (iii) 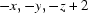; (iv) 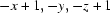; (v) 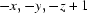.

**Fig. 1.**
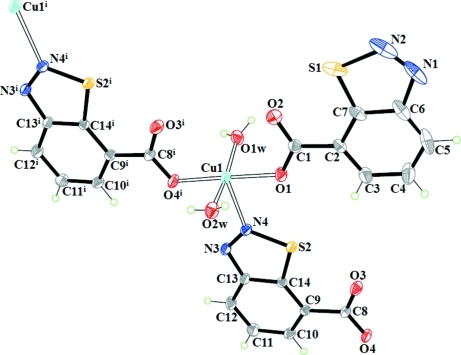
The molecular structure of the title molecule with the atom-numbering scheme. Displacement ellipsoids are drawn at the 30% probability level [symmetry code: (i) x - 1 , y, z].

**Fig. 2.**
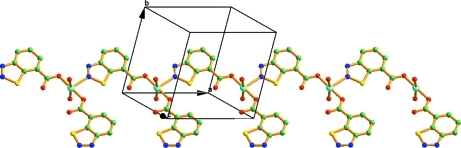
A partial packing diagram.

## supplementary crystallographic information

### Comment

The design and syntheses of supramolecular complexes exhibiting novel structures and their properties have provided exciting new prospects for chemists (Wang *et al.*,  2008). To date, a number of monometallic extended inorganic-organic composite materials have been synthesized by the combination of organic spacers and inorganic metal salts (Lan *et al.*,  2009). The rational design and construction of complexes greatly depend on the judicious selection of the organic ligands and proper choice of metal centers. Among various ligands, the versatile carboxylic acid ligands exhibiting diverse coordination modes, have been well used in the preparations of various metal-organic complexes, which exhibit interesting applications as functional materials (Hou *et al.*, 2004). 1,2,3-Benzothiadiazole-7-carboxylic acid (H*L*) known as a disease-resistance activator of plant is a versatile carboxylic acid ligand containing N and S donors for the following reasons: (1) H*L* has a group –COOH and a thiadiazole ring, in which the O, N and S atoms all could coordinate to metal ions. So H*L* can act as a bridging ligand. (2) The large conjugated π system of benzothiadiazole ring may act as the directing group for π···π stacking and C—H···π interactions. (3) Both N and O atoms in H*L* being typical electron donor in forming hydrogen bond, therefore may construct H-bonded supramolecular framework. However, up to now, H*L* has been largely ignored by coordination chemists and it has not been used for the preparation of metal complexes. We report herein the preparation and crystal structure of the title complex.

In the polymeric title complex, each Cu atom is surrounded by three 1,2,3-benzo- thiadiazole-7-carboxylate and two water molecules. A 1,2,3-benzothiadiazole-7 -carboxylate ligand bridges the two Cu atoms (Fig. 1) with a Cu···Cu distance of 9.006 (2) Å. The four O atoms (O1, O1W, O2W and O4^i^) [symmetry code (i): x - 1, y, z] in the equatorial planes around each Cu atom form a distorted square-planar arrangement, while the distorted square-pyramidal coordination is completed by the symmetry related N atoms of the bridging 1,2,3-benzothiadiazole -7-carboxylate ligands (Fig. 2).

In general, several parameters are often used to define the coordination geometries of the penta-coordinated metal centers, and one of the most common parameters is the τ factor defined by Addison *et al.* (τ = 0 for perfect square-pyramid environment and τ = 1 for trigonal bipyramidal geometry) (Addison *et al.*,  1984). For the title compound, the calculated τ value is 0.173, which corresponds to an approximately square-pyramidal coordination environment.

The Cu-O and Cu-N bonds (Table 1) are in good agreement with the coreesponding values reported for Cu-carboxylate, [Cu(H_2_O)_2_], and Cu-thiadiazole complexes (Lukashuk *et al.*,  2007; Qin *et al.*,  2009). To the best of our knowledge, there is no report on the crystal structures of metal complexes of 1,2,3-thiadiazole, except of a molybdenum pentacarbonyl complex of 4-phenyl-1,2,3-thiadiazole (Batzel & Boese, 1981) and a silver tetrafluoroborate complex of 2-(1,2,3-thiadiazol-4-yl)pyridine (Richardson & Steel, 2002).

In the crystal structure, strong intermolecular O-H···O and O-H···N hydrogen bonds (Table 2) link the molecules into a three-dimensional supramolecular network, in which they may be effective in the stabilization of the structure.

### Experimental

1,2,3-Benzothiadiazole-7-carboxylic acid (H*L*) was prepared according to the method of Fan *et al.* (2005) and Walter & Beat (1997). For the preparation of the title complex, a mixture of cupric nitrate (0.233 g, 1 mmol), H*L* (0.090 g, 0.5 mmol), sodium azide (0.065 g, 1 mmol) and water (12 ml) were placed in a Teflon reactor (23 ml), which was heated to 413 K for 2 d, and then cooled to room temperature at a rate of 5 K h^-1^. The crystals obtained were washed with water and dried in air (yield; 0.034 g, 30%).

### Refinement

H atoms were positioned geometrically with O-H = 0.85 Å (for H_2_O) and C-H = 0.93 Å for aromatic H atoms, respectively, and constrained to ride on their parent atoms, with U_iso_(H) = 1.2U_eq_(C,O).

### Crystal data

**Table:**

[Cu(C_7_H_3_N_2_O_2_S)_2_(H_2_O)_2_]	*Z* = 2
*M**_r_* = 457.95	*F*(000) = 462
Triclinic, *P*1	*D*_x_ = 1.725 Mg m^−^^3^
Hall symbol: -P 1	Mo *K*α radiation, λ = 0.71073 Å
*a* = 9.0061 (18) Å	Cell parameters from 8567 reflections
*b* = 9.4989 (19) Å	θ = 3.0–27.5°
*c* = 11.274 (2) Å	µ = 1.52 mm^−^^1^
α = 86.62 (3)°	*T* = 294 K
β = 70.00 (3)°	Block, green
γ = 76.69 (3)°	0.28 × 0.26 × 0.24 mm
*V* = 881.8 (4) Å^3^	

### Data collection

**Table:**

Rigaku R-AXIS RAPID-S diffractometer	3089 independent reflections
Radiation source: fine-focus sealed tube	2767 reflections with *I* > 2σ(*I*)
graphite	*R*_int_ = 0.027
ω scans	θ_max_ = 25.0°, θ_min_ = 3.0°
Absorption correction: multi-scan (*SADABS*; Bruker, 1998)	*h* = −10→10
*T*_min_ = 0.676, *T*_max_ = 0.712	*k* = −11→11
7632 measured reflections	*l* = −13→13

### Refinement

**Table:**

Refinement on *F*^2^	Primary atom site location: structure-invariant direct methods
Least-squares matrix: full	Secondary atom site location: difference Fourier map
*R*[*F*^2^ > 2σ(*F*^2^)] = 0.036	Hydrogen site location: inferred from neighbouring sites
*wR*(*F*^2^) = 0.083	H-atom parameters constrained
*S* = 1.08	*w* = 1/[σ^2^(*F*_o_^2^) + (0.0321*P*)^2^ + 0.8111*P*] where *P* = (*F*_o_^2^ + 2*F*_c_^2^)/3
3089 reflections	(Δ/σ)_max_ = 0.001
244 parameters	Δρ_max_ = 0.72 e Å^−^^3^
0 restraints	Δρ_min_ = −0.70 e Å^−^^3^

### Special details

**Table:**

Geometry. All e.s.d.'s (except the e.s.d. in the dihedral angle between two l.s. planes) are estimated using the full covariance matrix. The cell e.s.d.'s are taken into account individually in the estimation of e.s.d.'s in distances, angles and torsion angles; correlations between e.s.d.'s in cell parameters are only used when they are defined by crystal symmetry. An approximate (isotropic) treatment of cell e.s.d.'s is used for estimating e.s.d.'s involving l.s. planes.
Refinement. Refinement of *F*^2^ against ALL reflections. The weighted *R*-factor *wR* and goodness of fit *S* are based on *F*^2^, conventional *R*-factors *R* are based on *F*, with *F* set to zero for negative *F*^2^. The threshold expression of *F*^2^ > σ(*F*^2^) is used only for calculating *R*-factors(gt) *etc*. and is not relevant to the choice of reflections for refinement. *R*-factors based on *F*^2^ are statistically about twice as large as those based on *F*, and *R*- factors based on ALL data will be even larger.

### Fractional atomic coordinates and isotropic or equivalent isotropic displacement parameters (Å^2^)

**Table:**

	*x*	*y*	*z*	*U*_iso_*/*U*_eq_	
Cu1	0.07536 (4)	0.21613 (4)	0.52609 (3)	0.03039 (13)	
S1	−0.01189 (13)	−0.19206 (11)	0.93629 (9)	0.0624 (3)	
S2	0.46168 (8)	0.25680 (7)	0.48652 (7)	0.02732 (17)	
O1	0.1924 (2)	0.1114 (2)	0.6325 (2)	0.0383 (5)	
O2	−0.0035 (3)	−0.0020 (3)	0.7417 (2)	0.0503 (6)	
O3	0.7573 (2)	0.2143 (2)	0.5117 (2)	0.0394 (5)	
O4	0.9626 (2)	0.3236 (2)	0.41845 (19)	0.0342 (5)	
O1W	−0.0550 (3)	0.3574 (2)	0.6674 (2)	0.0419 (5)	
H1WA	−0.1223	0.4295	0.6535	0.050*	
H1WB	−0.0840	0.3276	0.7426	0.050*	
O2W	0.1701 (2)	0.0527 (2)	0.4022 (2)	0.0392 (5)	
H2WA	0.1911	−0.0281	0.4364	0.047*	
H2WB	0.1169	0.0445	0.3546	0.047*	
N1	0.1677 (6)	−0.2901 (4)	1.0612 (3)	0.0790 (12)	
N2	0.0307 (6)	−0.2940 (4)	1.0531 (3)	0.0850 (13)	
N3	0.3144 (3)	0.4182 (3)	0.3579 (2)	0.0317 (6)	
N4	0.2960 (3)	0.3209 (3)	0.4435 (2)	0.0297 (5)	
C1	0.1326 (4)	0.0272 (3)	0.7175 (3)	0.0338 (7)	
C2	0.2317 (4)	−0.0441 (3)	0.7958 (3)	0.0325 (7)	
C3	0.3828 (4)	−0.0218 (4)	0.7812 (3)	0.0449 (8)	
H3	0.4279	0.0396	0.7189	0.054*	
C4	0.4696 (5)	−0.0901 (5)	0.8587 (4)	0.0679 (12)	
H4	0.5723	−0.0749	0.8461	0.081*	
C5	0.4045 (7)	−0.1796 (5)	0.9534 (4)	0.0801 (14)	
H5	0.4615	−0.2231	1.0058	0.096*	
C6	0.2523 (6)	−0.2038 (4)	0.9695 (3)	0.0589 (11)	
C7	0.1684 (4)	−0.1379 (3)	0.8911 (3)	0.0417 (8)	
C8	0.8229 (3)	0.3009 (3)	0.4353 (3)	0.0280 (6)	
C9	0.7259 (3)	0.3865 (3)	0.3602 (3)	0.0248 (6)	
C10	0.7796 (3)	0.4848 (3)	0.2697 (3)	0.0324 (7)	
H10	0.8837	0.4994	0.2516	0.039*	
C11	0.6799 (4)	0.5634 (4)	0.2040 (3)	0.0417 (8)	
H11	0.7201	0.6279	0.1427	0.050*	
C12	0.5250 (4)	0.5467 (4)	0.2289 (3)	0.0408 (8)	
H12	0.4596	0.5999	0.1861	0.049*	
C13	0.4677 (3)	0.4475 (3)	0.3204 (3)	0.0290 (6)	
C14	0.5674 (3)	0.3674 (3)	0.3842 (2)	0.0235 (6)	

### Atomic displacement parameters (Å^2^)

**Table:**

	*U*^11^	*U*^22^	*U*^33^	*U*^12^	*U*^13^	*U*^23^
Cu1	0.0250 (2)	0.0314 (2)	0.0390 (2)	−0.00883 (15)	−0.01637 (16)	0.01192 (16)
S1	0.0723 (7)	0.0527 (6)	0.0441 (5)	−0.0254 (5)	0.0109 (5)	−0.0026 (4)
S2	0.0229 (4)	0.0284 (4)	0.0320 (4)	−0.0074 (3)	−0.0111 (3)	0.0081 (3)
O1	0.0362 (12)	0.0411 (12)	0.0418 (12)	−0.0105 (10)	−0.0200 (10)	0.0181 (10)
O2	0.0419 (14)	0.0670 (16)	0.0460 (14)	−0.0204 (12)	−0.0153 (11)	0.0068 (12)
O3	0.0299 (11)	0.0369 (12)	0.0539 (14)	−0.0090 (9)	−0.0190 (10)	0.0187 (11)
O4	0.0251 (10)	0.0385 (11)	0.0447 (12)	−0.0079 (9)	−0.0196 (9)	0.0095 (10)
O1W	0.0441 (13)	0.0423 (12)	0.0357 (12)	0.0003 (10)	−0.0170 (10)	0.0092 (10)
O2W	0.0419 (12)	0.0311 (11)	0.0498 (13)	−0.0092 (9)	−0.0229 (11)	0.0104 (10)
N1	0.144 (4)	0.056 (2)	0.0344 (18)	−0.032 (3)	−0.024 (2)	0.0204 (16)
N2	0.134 (4)	0.064 (2)	0.0390 (19)	−0.038 (3)	0.002 (2)	0.0074 (17)
N3	0.0251 (12)	0.0383 (14)	0.0354 (14)	−0.0095 (11)	−0.0147 (11)	0.0091 (11)
N4	0.0234 (12)	0.0335 (13)	0.0339 (13)	−0.0091 (10)	−0.0111 (11)	0.0071 (11)
C1	0.0348 (17)	0.0346 (16)	0.0311 (16)	−0.0053 (14)	−0.0110 (14)	−0.0020 (13)
C2	0.0415 (18)	0.0295 (15)	0.0229 (14)	−0.0044 (13)	−0.0087 (13)	0.0009 (12)
C3	0.052 (2)	0.047 (2)	0.0402 (19)	−0.0129 (16)	−0.0223 (17)	0.0093 (16)
C4	0.070 (3)	0.079 (3)	0.073 (3)	−0.018 (2)	−0.050 (2)	0.014 (2)
C5	0.122 (4)	0.073 (3)	0.066 (3)	−0.020 (3)	−0.065 (3)	0.031 (2)
C6	0.098 (3)	0.046 (2)	0.0348 (19)	−0.015 (2)	−0.027 (2)	0.0093 (17)
C7	0.060 (2)	0.0334 (16)	0.0248 (15)	−0.0096 (15)	−0.0057 (15)	−0.0015 (13)
C8	0.0238 (14)	0.0255 (14)	0.0340 (16)	−0.0019 (12)	−0.0108 (12)	−0.0004 (12)
C9	0.0224 (14)	0.0237 (14)	0.0277 (14)	−0.0036 (11)	−0.0083 (12)	−0.0030 (11)
C10	0.0263 (15)	0.0358 (16)	0.0359 (16)	−0.0114 (13)	−0.0092 (13)	0.0048 (13)
C11	0.0410 (18)	0.0477 (19)	0.0409 (18)	−0.0204 (15)	−0.0159 (15)	0.0224 (16)
C12	0.0376 (18)	0.0481 (19)	0.0421 (18)	−0.0128 (15)	−0.0215 (15)	0.0221 (15)
C13	0.0240 (14)	0.0338 (15)	0.0323 (15)	−0.0083 (12)	−0.0132 (13)	0.0060 (13)
C14	0.0232 (14)	0.0232 (13)	0.0234 (13)	−0.0047 (11)	−0.0075 (11)	0.0003 (11)

### Geometric parameters (Å, °)

**Table:**

Cu1—O4^i^	1.9405 (19)	C4—H4	0.9300
Cu1—O1	1.945 (2)	C5—C6	1.391 (6)
Cu1—O1W	1.991 (2)	C5—H5	0.9300
Cu1—O2W	1.980 (2)	C6—C7	1.385 (5)
Cu1—N4	2.311 (2)	C6—N1	1.404 (5)
O1W—H1WA	0.8500	C7—S1	1.717 (4)
O1W—H1WB	0.8500	C8—O3	1.249 (3)
O2W—H2WA	0.8500	C8—O4	1.273 (3)
O2W—H2WB	0.8500	C8—C9	1.497 (4)
N1—N2	1.277 (6)	C9—C10	1.380 (4)
N2—S1	1.686 (4)	C9—C14	1.412 (4)
N3—N4	1.292 (3)	C10—C11	1.412 (4)
N4—S2	1.695 (2)	C10—H10	0.9300
C1—O2	1.254 (4)	C11—C12	1.371 (4)
C1—O1	1.265 (4)	C11—H11	0.9300
C1—C2	1.493 (4)	C12—C13	1.401 (4)
C2—C3	1.378 (4)	C12—H12	0.9300
C2—C7	1.405 (4)	C13—N3	1.388 (3)
C3—C4	1.400 (5)	C13—C14	1.402 (4)
C3—H3	0.9300	C14—S2	1.706 (3)
C4—C5	1.379 (6)		
			
O4^i^—Cu1—O1	178.57 (8)	C4—C3—H3	119.5
O4^i^—Cu1—O2W	90.50 (9)	C5—C4—C3	120.7 (4)
O1—Cu1—O2W	89.78 (9)	C5—C4—H4	119.7
O4^i^—Cu1—O1W	90.40 (9)	C3—C4—H4	119.7
O1—Cu1—O1W	89.61 (9)	C4—C5—C6	119.1 (4)
O2W—Cu1—O1W	168.13 (9)	C4—C5—H5	120.5
O4^i^—Cu1—N4	93.41 (8)	C6—C5—H5	120.5
O1—Cu1—N4	85.18 (8)	C7—C6—C5	119.9 (3)
O2W—Cu1—N4	93.52 (9)	C7—C6—N1	113.5 (4)
O1W—Cu1—N4	98.24 (9)	C5—C6—N1	126.7 (4)
N2—S1—C7	92.6 (2)	C6—C7—C2	121.7 (3)
N4—S2—C14	91.77 (12)	C6—C7—S1	107.5 (3)
C1—O1—Cu1	122.34 (19)	C2—C7—S1	130.8 (3)
C8—O4—Cu1^ii^	115.52 (18)	O3—C8—O4	125.5 (3)
Cu1—O1W—H1WA	118.0	O3—C8—C9	116.4 (2)
Cu1—O1W—H1WB	119.4	O4—C8—C9	118.1 (2)
H1WA—O1W—H1WB	114.1	C10—C9—C14	117.3 (2)
Cu1—O2W—H2WA	112.6	C10—C9—C8	124.6 (2)
Cu1—O2W—H2WB	116.3	C14—C9—C8	118.1 (2)
H2WA—O2W—H2WB	108.7	C9—C10—C11	121.4 (3)
N2—N1—C6	113.2 (4)	C9—C10—H10	119.3
N1—N2—S1	113.3 (3)	C11—C10—H10	119.3
N4—N3—C13	112.3 (2)	C12—C11—C10	121.3 (3)
N3—N4—S2	114.07 (18)	C12—C11—H11	119.3
N3—N4—Cu1	126.89 (17)	C10—C11—H11	119.3
S2—N4—Cu1	118.78 (12)	C11—C12—C13	118.4 (3)
O2—C1—O1	125.6 (3)	C11—C12—H12	120.8
O2—C1—C2	116.8 (3)	C13—C12—H12	120.8
O1—C1—C2	117.6 (3)	N3—C13—C12	126.0 (3)
C3—C2—C7	117.5 (3)	N3—C13—C14	113.7 (2)
C3—C2—C1	123.5 (3)	C12—C13—C14	120.4 (2)
C7—C2—C1	118.9 (3)	C13—C14—C9	121.2 (2)
C2—C3—C4	121.1 (3)	C13—C14—S2	108.20 (19)
C2—C3—H3	119.5	C9—C14—S2	130.6 (2)
			
O2—C1—C2—C3	−179.4 (3)	C8—C9—C14—C13	177.9 (2)
O1—C1—C2—C3	0.7 (4)	C10—C9—C14—S2	179.5 (2)
O2—C1—C2—C7	−0.2 (4)	C8—C9—C14—S2	−1.2 (4)
O1—C1—C2—C7	179.9 (3)	C7—C6—N1—N2	1.4 (5)
C7—C2—C3—C4	−0.1 (5)	C5—C6—N1—N2	−179.3 (4)
C1—C2—C3—C4	179.1 (3)	C6—N1—N2—S1	−1.0 (5)
C2—C3—C4—C5	−1.2 (6)	C12—C13—N3—N4	179.6 (3)
C3—C4—C5—C6	1.4 (7)	C14—C13—N3—N4	−0.8 (4)
C4—C5—C6—C7	−0.2 (6)	C13—N3—N4—S2	0.2 (3)
C4—C5—C6—N1	−179.4 (4)	C13—N3—N4—Cu1	−173.94 (19)
C5—C6—C7—C2	−1.1 (6)	O4^i^—Cu1—N4—N3	1.4 (2)
N1—C6—C7—C2	178.2 (3)	O1—Cu1—N4—N3	−178.4 (2)
C5—C6—C7—S1	179.6 (3)	O2W—Cu1—N4—N3	92.1 (2)
N1—C6—C7—S1	−1.1 (4)	O1W—Cu1—N4—N3	−89.5 (2)
C3—C2—C7—C6	1.2 (5)	O4^i^—Cu1—N4—S2	−172.46 (14)
C1—C2—C7—C6	−178.0 (3)	O1—Cu1—N4—S2	7.74 (14)
C3—C2—C7—S1	−179.6 (3)	O2W—Cu1—N4—S2	−81.75 (14)
C1—C2—C7—S1	1.2 (4)	O1W—Cu1—N4—S2	96.64 (14)
O3—C8—C9—C10	−178.6 (3)	O2—C1—O1—Cu1	1.9 (4)
O4—C8—C9—C10	2.8 (4)	C2—C1—O1—Cu1	−178.27 (19)
O3—C8—C9—C14	2.2 (4)	O2W—Cu1—O1—C1	−90.3 (2)
O4—C8—C9—C14	−176.4 (2)	O1W—Cu1—O1—C1	77.8 (2)
C14—C9—C10—C11	0.3 (4)	N4—Cu1—O1—C1	176.1 (2)
C8—C9—C10—C11	−179.0 (3)	O3—C8—O4—Cu1^ii^	−1.8 (4)
C9—C10—C11—C12	0.9 (5)	C9—C8—O4—Cu1^ii^	176.67 (18)
C10—C11—C12—C13	−0.9 (5)	N1—N2—S1—C7	0.3 (4)
C11—C12—C13—N3	179.4 (3)	C6—C7—S1—N2	0.5 (3)
C11—C12—C13—C14	−0.2 (5)	C2—C7—S1—N2	−178.8 (3)
N3—C13—C14—C9	−178.2 (2)	N3—N4—S2—C14	0.4 (2)
C12—C13—C14—C9	1.4 (4)	Cu1—N4—S2—C14	175.00 (14)
N3—C13—C14—S2	1.0 (3)	C13—C14—S2—N4	−0.8 (2)
C12—C13—C14—S2	−179.3 (2)	C9—C14—S2—N4	178.4 (3)
C10—C9—C14—C13	−1.4 (4)		

Symmetry codes: (i) *x*−1, *y*, *z*; (ii) *x*+1, *y*, *z*.

### Hydrogen-bond geometry (Å, °)

**Table:**

*D*—H···*A*	*D*—H	H···*A*	*D*···*A*	*D*—H···*A*
O1w—H1wA···N3^iii^	0.85	2.02	2.859 (4)	169
O1w—H1wB···N1^iv^	0.85	2.12	2.957 (4)	170
O2w—H2wA···O3^v^	0.85	1.84	2.680 (3)	172
O2w—H2wB···O2^vi^	0.85	1.84	2.684 (3)	172

Symmetry codes: (iii) −*x*, −*y*+1, −*z*+1; (iv) −*x*, −*y*, −*z*+2; (v) −*x*+1, −*y*, −*z*+1; (vi) −*x*, −*y*, −*z*+1.
